# A comparative analysis of aerosol exposure and prevention strategies in bystander, pre-hospital, and inpatient cardiopulmonary resuscitation using simulation manikins

**DOI:** 10.1038/s41598-023-39726-x

**Published:** 2023-08-02

**Authors:** Tzu-Yao Hung, Chung-Shiung Wen, Sheng-Han Yu, Yi-Chang Chen, Hsin-Ling Chen, Wei-Lun Chen, Chih-Chieh Wu, Yung-Cheng Su, Chun-Lung Lin, Shih-Cheng Hu, Tee Lin

**Affiliations:** 1 Department of Emergency Medicine, Zhong-Xing Branch, Taipei City Hospital, Taipei City, Taiwan; 2 School of Medicine, National Yang Ming Chiao Tung University, Taipei, Taiwan; 3 CrazyatLAB (Critical Airway Training Laboratory), Taipei City, Taiwan; 4 Department of Energy and Refrigerating Air-Conditioning Engineering, National Taipei University of Technology, Taipei, Taiwan; 5 School of Medicine, Tzu Chi University, Hualien County, Taiwan; 6 Department of Emergency Medicine, Ditmanson Medical Foundation Chiayi Christian Hospital, No.539, Zhongxiao Rd., East Dist., Chiayi City, 600566 Taiwan

**Keywords:** Health care, Infectious diseases

## Abstract

To evaluate aerosol exposure risk and prevention strategies during bystander, pre-hospital, and inpatient cardiopulmonary resuscitation (CPR). This study compared hands-only CPR, CPR with a surgical or N95 mask, and CPR with a non-rebreather mask at 15 L/min. 30:2 compression–ventilation ratio CPR was tested with face-mask ventilation (FMV), FMV with a high efficiency particulate air (HEPA) filter; supraglottic airway (SGA), SGA with a surgical mask, SGA with a HEPA filter, or SGA with both. Continuous CPR was tested with an endotracheal tube (ET), ET with a surgical mask, a HEPA filter, or both. Aerosol concentration at the head, trunk, and feet of the mannequin were measured to evaluate exposure to CPR personnel. Hands-only CPR with a surgical or N95 face mask coverings and ET tube ventilation CPR with filters showed the lowest aerosol exposure among all study groups, including CPR with NRM oxygenation, FMV, and SGA ventilation. NRM had a mask effect and reduced aerosol exposure at the head, trunk, and feet of the mannequin. FMV with filters during 30:2 CPR reduced aerosol exposure at the head and trunk, but increased at the feet of the mannequin. A tightly-sealed SGA when used with a HEPA filter, reduced aerosol exposure by 21.00–63.14% compared with a loose-fitting one. Hands-only CPR with a proper fit surgical or N95 face mask coverings is as safe as ET tube ventilation CPR with filters, compared with CPR with NRM, FMV, and SGA. FMV or tight-sealed SGA ventilation with filters prolonged the duration to achieve estimated infective dose of SARS-CoV-2 2.4–2.5 times longer than hands-on CPR only. However, a loose-fitting SGA is not protective at all to chest compressor or health workers standing at the foot side of the victim, so should be used with caution even when using with HEPA filters.

## Introduction

Severe acute respiratory syndrome coronavirus 2 (SARS-CoV-2) is a single-stranded RNA virus, first reported in Wuhan, China, in December 2019 that quickly spread across the globe. The virus infected more than 60 billion people worldwide and from September 2020 to present has led to over 6.4 million deaths^1^. In addition, an estimated 14.9 million excess deaths occurred during 2020 and 2021^2^. Multiple waves during the pandemic caused by different variants of SARS-CoV-2 has placed resource constraints on health care systems^3^. During the pandemic period, higher rates of out-of-hospital cardiac arrest (OHCA)^4–6^, less bystander cardiopulmonary resuscitation^4,6–9^, and lower rates of initial shockable rhythm^4–6,9^, delays in resuscitation^4,6,8,9^, and lower survival rates of OHCA patients with return of spontaneous circulation before admission^4–7,9^ were observed in the pre-hospital setting. In the inpatient setting during the pandemic, a higher incidence of in-hospital cardiac arrest (IHCA) and lower rates of shockable rhythm^10,11^ was observed, but overall survival was similar to pre-pandemic levels or lower, depending on the regional hospital burden^10,11^.

Early cardiopulmonary resuscitation (CPR) and defibrillation improve survival in OHCA^12^. One review reported decreased rates of bystander CPR in Europe after the start of the pandemic but no significant differences in the US^9,13^. The concern of COVID transmission reduced the willingness of bystanders to administer CPR^9,13,14^. COVID is most commonly transmitted through droplets, aerosols (diameter of ≤ 5 µm), and fomites^15–17^. Several essential procedures during outpatient and inpatient resuscitation are considered aerosol generating procedures: chest compression, pre-oxygenation, and manual ventilation^16,18,19^.

The main risk during CPR is the constant chest compression expelling the viral aerosols from the airway system of the cardiac arrest victim with COVID-19. The dispersion of aerosols during each chest compression is not much compared with active breathing, however, 100–120 compressions per minute is far more than the respiration rate of a human. The longer the duration of CPR, the higher concentration of aerosol exposure would be resulted. A passive reverse flow was detectable during each chest compression and the mean volume was 7.5–41.5 mL^20–22^. Consensus statements from multiple committees agree on using lower aerosolization risk strategies during chest compressions: cover the victim’s mouth with a face mask or cloth during basic life support; use a non-rebreather mask for adult CPR before advanced airway placement; avoid poor-fitting bag mask ventilation; use only experienced airway staff to administer supraglottic airway (SGA) or endotracheal tube (ET) in order to maintain the airway, and do so earlier in the resuscitation; connect a high-efficiency particulate air (HEPA) filter securely to the mask, SGA, or ET before there is any chance for passive exhaled breath from the victim during CPR^23–26^.

Mostafa et al. tested second-generation SGAs in another simulation study using powder which glows under ultraviolet light^27^. They concluded ET with a HEPA filter was effective in reducing aerosol dispersion, but SGAs with a HEPA filter were still aerosol-generating, regardless of the type of SGA^28^. Although these visualization studies are impressive, the real-world aerosol concentration and potential for exposure at the head (ventilation personnel), the trunk (chest compression personnel), and the assistant at the feet during CPR remain unknown.

What is the impact of current aerosol prevention strategies during CPR on mitigating the risks of aerosol exposure for medical personnel, particularly in a time when standard personal protective equipment is no longer routinely used? Additionally, the investigation of aerosols during CPR presents moral controversy. Smith et al. had studied dynamic modeling of exhaled respiratory droplets via tracer gas of 1% glycerol to evaluate the aerosol transmission^29^. We adopted this model and conducted this investigation to evaluate the effectiveness of current strategies to reduce aerosol transmission during CPR.

## Methods

### Study design and setting

**Figure 1:**
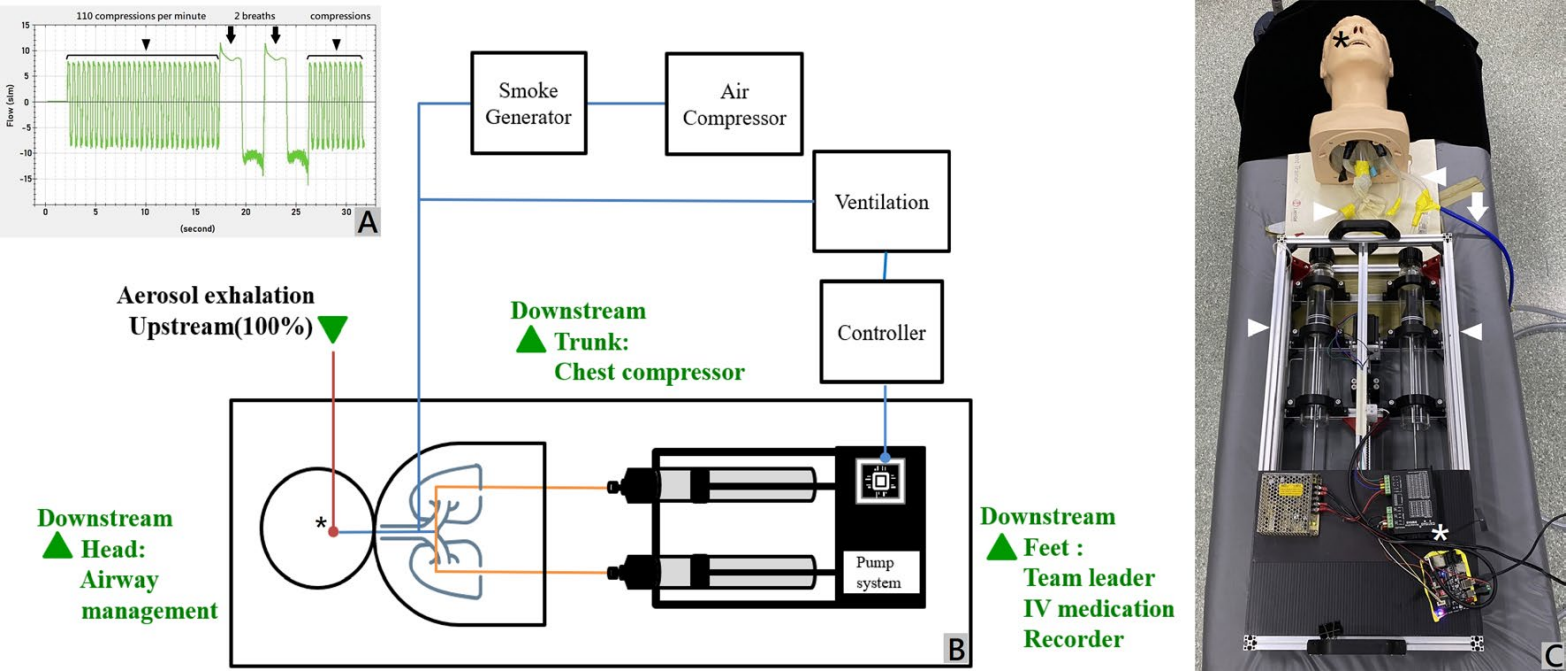
(**A**) Two modes of simulation were conducted in the study: continuous compression and 30:2 compression–ventilation ratio CPR. The compression speed was 110 compressions per minute (black arrowhead), which created a reversed flow of 23.1 mL during each compression. At the 30:2 compression–ventilation mode, there were a pause period lasting 8 s for two ventilations, 0.5 L each. (**B**) The diagram for aerosol concentration setting. Poly-alpha-olefin (PAO) was used as a tracer gas. The aerosol exhaled from the mouth of the mannequin was defined as upstream and 100% (asterisk). Aerosol concentrations were measured with a light-scattering photometer at three spots (downstream at the head, trunk, and feet of the mannequin) to detect the aerosol exposure of personnel who performed airway management, chest compressions, and for the medication giver, recorder, or team leader. The pump system was controlled by a central processing unit for continuous and synchronized 30:2 CPR. (**C**) The pump system was controlled by a central processing unit (white asterisk) and connected to the mannequin’s airway (white arrowheads). The tracer gas at an aerosol level of 0.5–0.7 μm generated by the air compressor (the white arrow and the blue tube) was sucked in and compressed out via the mannequin’s mouth (black asterisk).

This simulation study was exempted from the Taipei City Hospital Research Ethics Committee. The study was conducted at the resuscitation room of Taipei City Hospital, ZhongXing branch, Taipei, Taiwan. The resuscitation room has a downward background flow with air flowing from the top to the four vents at the bottom corners. The ventilation system ensures a rate of 12 air changes per hour. The room temperature was 21.1 ± 0.2℃. The relative humidity was 38.1 ± 2.0%. A high-fidelity simulation mannequin (Airway Management Trainer, Laerdal®, Norway) was used, and its airway was connected to a smoke particle generator (MPL-I003, Tong-Da industry company, Taiwan). A pump system controlled by a central processing unit provided both continuous and compression-ventilation ratios of 30:2 (Fig. 1A–C). The 30:2 mode in the setup delivers 30 cycles of reversed flow, maintaining the same exhaled amount and rate as the continuous mode (23.1 mL exhalation and 110 cycles per minute). However, it incorporates an 8-s pause, during which two 500 mL ventilations are compressed into the mannequin’s airway. This simulation is designed to replicate synchronized CPR in accordance with the 2020 advanced cardiac lift support (ACLS) guidelines^24^ (Fig. 1A). Atomized poly-alpha-olefin, with a diameter of 0.5–0.7 μm, was used as a tracer aerosol gas and was measured at the head, trunk, and feet around the mannequin with a light-scattering photometer at a sampling rate of 28.3 L/min over 10 min. The mouth of the mannequin was defined as upstream (100%), and three locations, defined as downstream (head, trunk, and feet), were monitored for aerosol concentrations (Fig. 1B).

**Figure 2:**
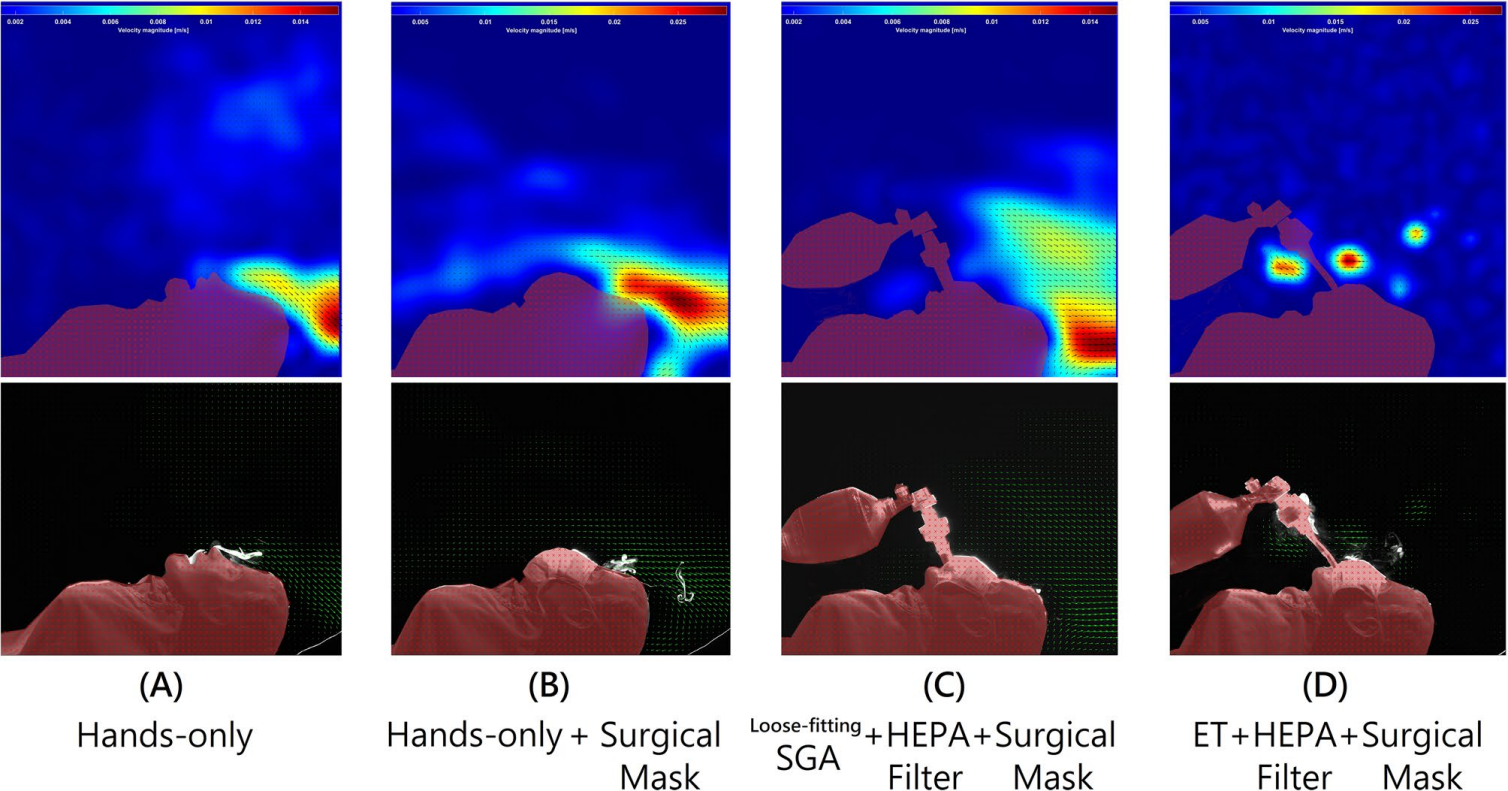
Aerosol movement with velocity vector (green arrows) under particle image velocimetry. Note the prominent aerosol dispersion with loose-fitting SGA.

Glycerol was used as a tracer gas during the large-scale particle image velocimetry (PIV) and recorded using a high-sensitivity camera (ORCA-Flash 4.0 V2 digital CMOS camera, Hamamatsu Co., Japan) to identify the direction of the airflow in the space^19,29^ (Fig. 2). Both aerosol concentration and PIV results were reviewed for consistency of airflow movement in the space.

### Interventions

The reference setting was continuous CPR mode with no face covering to simulate hands-only CPR before the COVID era. Six 30:2 compression-ventilation ratio settings and seven continuous CPR settings were tested: face mask ventilation (FMV); FMV with HEPA filter; supraglottic airway (SGA), SGA with face covering, SGA with HEPA filter, SGA with face covering and HEPA filter; continuous CPR with surgical mask covering, continuous CPR with N95 mask covering (successfully passed the quantitative fit test), non-rebreather mask at 15 L/min with face covering, continuous CPR with endotracheal intubation, continuous CPR with ET with face covering, continuous CPR with ET with HEPA filter, and continuous CPR with ET with face covering and HEPA filter. 30:2 compression-ventilation ratio CPR was tested again with a loose-fitting SGA (1 mm away from the proper SGA position) with HEPA filter, a loose-fitting SGA with face covering and HEPA filter. In total, fifteen test groups were measured and compared with the reference setting for aerosol exposure. The SGA (Size 4, Ambu® Aura-i™, Denmark) was appropriately positioned using a video stylet to ensure proper adherence between the SGA and the glottis. The ET tube, with a size of 7.5 mm, was sourced from Rüsch® (Teleflex, United States). The cuff was inflated with 10 mL of air.

### Measurements

The particle in velocity visualization flow field in the resuscitation room was recorded using a high sensitivity camera under a green laser radiation plane and was analyzed for flow direction and presented as vector graphs.

Next, aerosol concentrations at the head, trunk, and feet of the mannequin were measured continuously over 10 min with the thirteen intervention subgroups and compared to the reference group. Between measurements, detection began only after the aerosol concentration fell below the baseline (50 ppm).

### Outcomes

The primary outcome was the aerosol concentration at the head, trunk, and feet of the mannequin. The visualized particle in velocity was also analyzed to trace the airflow movement in the space. The estimated accumulation time to achieve the number of particles to infect an individual (Nf) was calculated to be more conceptualized to realize the study result. The estimated accumulation time was based on the emission rate of particles from a live patient with COVID-19, which can range from 1000 to 100,000 particles per minute^29^.

### Analysis

Analyses were performed using the SAS System for Unix, version 9.4 (SAS Institute, Inc., Cary, NC, USA) and STATA software, version 15.1 (StataCorp, College Station, TX, USA). The mean percentage differences were evaluated using Student’s *t* tests among each setting of CPR. In total 48 comparisons—16 × 3 possibilities—were analyzed involving one reference group of hands-only CPR and thirteen experiment groups at the head, trunk, and feet around the mannequin. We used the Bonferroni correction to adjust for multiple testing issues and address the multiple comparisons problem. Given the substantial number of multiple tests (48 comparisons), we adjusted the confidence interval to 99.9%. To detect a mean percentage difference of 20% from the reference baseline with a two-sided significance level of 0.001 and 80% power, we estimated that 1251 participants would be required for each subgroup tested. We measured each study group and the reference group 1500 times for a duration of 10 min at the mannequin’s head, trunk, and feet to evaluate aerosol exposure.

### Ethical approval

No human subjects were involved in this study. All methods were carried out in accordance with relevant guidelines and regulations. No need for informed consent due to no human subjects were involved. This simulation study was exempted from the Taipei City Hospital Research Ethics Committee (TCHIRB-11112002-W).

## Results

The mean ± standard deviations (SD) of the aerosol concentration for the reference group at the head, trunk, and feet were 2257.37 ± 3970.83, 874.82 ± 1508.90, 744.43 ± 363.80 ppm, respectively. All subgroups with face coverings or airway devices reduced the aerosol exposure at the head (Table 1). During the hands-only continuous CPR, aerosol concentrations were prominently reduced with the proper-fitting surgical mask and N95 mask at the head, compared to the reference group without a face covering, with means ± SD of 396.21 ± 547.46, 375.30 ± 263.07 at the head; 399.01 ± 154.76, 190.84 ± 76.38 at the trunk, 402.66 ± 173.49, 296.50 ± 308.56 ppm at the feet. Hands-only CPR with non-rebreather mask (NRM) at a flow rate of 15L/min revealed mask effect and reduced aerosol exposure at the head, trunk, and feet around the mannequin (785.39 ± 516.95, 623.79 ± 270.98, and 508.93 ± 196.91 ppm, respectively) (Table 1).

**Table 1:** Aerosol concentrations in a 10 min period were measured at the head, trunk, and feet of the mannequin to evaluate aerosol exposure for personnel who manage the airway, conduct chest compressions and defibrillation, and give intravenous medications or lead the CPR.

	NRM at 15L/min	Surgical mask	N95 mask	HEPA Filter	FMV	SGA	Mean (ppm)		Standard Deviation	99.9% CI of Mean Difference	Nf = 10 (min)	Nf = 20 (min)	Nf = 100 (min)
Head													
Hands-only continuous CPR							2257.37	±	3970.83	Reference	1.74	3.49	17.43
		✓					396.21	±	547.46	1519.95 − 2202.36	9.93	19.87	99.33
			✓				375.26	±	263.07	1543.34 − 2220.88	10.49	20.97	104.87
	✓						785.39	±	516.95	1131.12 − 1812.85	5.01	10.02	50.11
Compression–ventilation 30:2 CPR					✓		972.56	±	411.34	944.98 − 1624.64	4.05	8.09	40.46
				✓	✓		702.58	±	365.6	1215.34 − 1894.24	5.60	11.20	56.01
						✓	926.73	±	404.22	990.87 − 1670.41	4.25	8.49	42.47
		✓				✓	942.29	±	421.6	975.16 − 1654.99	4.18	8.35	41.76
				✓		*	908.76	±	409.39	1008.80 − 1688.42	4.33	8.66	43.31
		✓		✓		*	1020.16	±	765.11	893.00 − 1581.41	3.86	7.72	38.58
				✓		✓	717.89	±	1717.68	1171.37 − 1907.59	5.48	10.96	54.82
		✓		✓		✓	696.41	±	1138.84	1209.40 − 1912.52	5.65	11.30	56.51
ET with Continuous CPR							780.26	±	320.79	1137.98 − 1816.24	5.04	10.09	50.44
		✓					805.48	±	359.06	1112.49 − 1791.29	4.89	9.77	48.86
				✓			324.30	±	157.79	1594.775 − 2271.37	12.14	24.27	121.35
		✓		✓			213.31	±	108.00	1705.90 − 2382.22	18.45	36.90	184.49
Trunk													
Hands − only continuous CPR							874.82	±	1508.90	Reference	4.50	9.00	44.99
		✓					399.01	±	154.76	346.70 − 604.93	9.86	19.73	98.63
			✓				190.84	±	76.38	555.37 − 812.59	20.62	41.24	206.21
	✓						623.79	±	270.98	120.55 − 381.53	6.31	12.62	63.09
Compression − ventilation 30:2 CPR					✓		942.76	±	384.95	− 200.46 − 64.60	4.17	8.35	41.74
				✓	✓		732.05	±	350.33	10.93 − 274.61	5.38	10.75	53.76
						✓	749.36	±	341.98	− 6.23 − 257.14	5.25	10.50	52.52
		✓				✓	961.31	±	634.39	− 225.76 − 52.78	4.09	8.19	40.94
				✓		*	880.15	±	339.51	− 136.97 − 126.31	4.47	8.94	44.71
		✓		✓		*	981.86	±	776.33	− 251.40 − 37.32	4.01	8.02	40.08
				✓		✓	479.07	±	979.05	242.75 − 548.74	8.21	16.43	82.15
		✓		✓		✓	385.67	±	243	359.06 − 619.24	10.20	20.41	102.04
ET with Continuous CPR							767.18	±	328.17	− 23.79 − 239.07	5.13	10.26	51.30
		✓					768.19	±	319.26	− 24.64 − 237.91	5.12	10.25	51.23
				✓			300.96	±	142.8	444.84 − 702.88	13.08	26.15	130.76
		✓		✓			354.97	±	156.96	390.71 − 649.00	11.09	22.17	110.87
Feet													
Hands-only continuous CPR							744.43	±	363.80	Reference	5.29	10.57	52.86
		✓					402.66	±	173.49	307.48 − 376.06	9.77	19.55	97.74
			✓				296.50	±	308.56	407.37 − 488.51	13.27	26.55	132.73
	✓						508.93	±	196.91	200.31 − 270.70	7.73	15.47	77.33
Compression–ventilation 30:2 CPR					✓		1182.33	±	933.32	− 523.13 to − 352.66	3.33	6.66	33.29
				✓	✓		851.00	±	395.31	− 152.26 to − 60.88	4.62	9.25	46.24
						✓	1295.03	±	775.04	− 623.44 to − 477.76	3.04	6.08	30.39
		✓				✓	1307.24	±	804.83	− 637.95 to − 487.66	3.01	6.02	30.10
				✓		*	1300.08	±	679.48	− 621.21 to − 490.08	3.03	6.05	30.27
		✓		✓		*	1150.06	±	826.29	− 482.44 to − 328.81	3.42	6.84	34.22
				✓		✓	479.26	±	309.01	224.57 − 305.76	8.21	16.42	82.11
		✓		✓		✓	432.22	±	235.35	275.36 − 349.07	9.11	18.21	91.05
ET with Continuous CPR							1315.7	±	521.18	− 625.33 to − 517.21	2.99	5.98	29.91
		✓					1447.51	±	562.61	− 760.06 to − 646.09	2.72	5.44	27.19
				✓			348.8	±	229.02	359.07 − 432.20	11.28	22.57	112.83
		✓		✓			375.93	±	236.74	331.59 − 405.43	10.47	20.94	104.69

The number of measurements for each group were 1500 times. To translate the study result, the estimated accumulation time needs to achieve the number of particles to infect an individual (Nf) was calculated for each CPR subgroup at the head, trunk, and foot of the mannequin. The individual who participated the CPR was assumed to breath at a minute ventilation at 8L/min (tidal volume 0.5L × 16 inhalations/min). The emission rate of the alive patient with COVID-19 may range from 1000 to 100,000 particles/min^29^. The reversed exhaled flow for a minute of CPR was 23.1 mL × 110 times/minute, which was 2.54 L/min, equaled to 31.76% of an individual who breaths at 8L/min. The table was schemed at the emission rate of 317.6 (1000 × 31.76%) particles/min. The Nf of SARS-CoV-1 was 10–100 particles. The Nf of SARS-CoV-2 was unknown, however, was thought to be more efficient, thus caused the worldwide pandemic. CPR, Cardiopulmonary compression; ET, Endotracheal tube; NRM, Non-rebreather mask; HEPA, High efficiency particulate air; FMV, Face mask ventilation; SGA, Supraglottic airway.

*poor-sealed SGA; CI: confidence interval. The reference group was hands-only continuous CPR.

When comparing the 30:2 compression-ventilation ratio CPR and face mask ventilation (FMV) with the reference group, the FMV alone increased aerosols at the feet, but decreased aerosols at the head (Table 1). FMV with a High-Efficiency Particulate Air (HEPA) filter showed similar effect but the aerosol concentrations were reduced (FMV values were 972.56 ± 411.34, 942.76 ± 384.95, 1182.33 ± 933.32; FMV + HEPA filter values were 702.58 ± 365.60, 732.05 ± 350.33, 851.00 ± 395.31 ppm). The 30:2 CPR with a loose-fitting SGA and a HEPA filter significantly decreased the aerosol concentration at the head, but increased at the trunk and feet (908.76 ± 409.39, 880.15 ± 339.51, 1300.08 ± 679.48 ppm, respectively). However, 30:2 CPR with a tightly-sealed SGA and a HEPA filter, reduced the aerosol at the head, trunk, and feet, compared with the reference group (717.89 ± 1717.68, 479.07 ± 979.05, 479.26 ± 309.01 ppm, respectively) (Table 1).

When continuous CPR was conducted with endotracheal tube (ET) insertion and compared with the reference group, the ET alone or covered with a surgical mask both decreased aerosols at the head, but increased them at the trunk and feet (the means ± SDs were 780.26 ± 320.79 and 805.48 ± 359.06 at the head, 767.18 ± 328.17 and 768.19 ± 319.26 at the trunk, 1315.70 ± 521.18 and 1447.51 ± 562.61 at the feet, respectively). When continuous CPR was performed with ET and a HEPA filter, or with a HEPA filter and surgical mask covering, the aerosols at the head, trunk, and feet were all reduced significantly (means ± SDs for ET, HEPA filter, and HEPA with surgical mask of 324.30 ± 157.79 and 213.31 ± 108.00 at the head, 300.96 ± 142.80 and 354.97 ± 156.96 at the trunk; 348.80 ± 229.02 and 375.93 ± 236.74 ppm at the feet, respectively) (Table 1).

When evaluating the effectiveness of a surgical mask and N95 mask covering on hands-only CPR, both masks revealed significant reduction at the head, trunk, and feet of the mannequin (Table 1). The average aerosol exposures of face covering with surgical mask and N95 mask were similar to the ET with HEPA filter subgroup (Table 1). However, when using surgical mask covering as an adjunct with FMV, SGA, or ET insertion, surgical masks cannot decrease the aerosol exposure (Table 2).

**Table 2:** The aerosol prevention strategies were compared with without for the reduction rate of aerosol exposure.

			Mean (ppm)		Standard Deviation	Mean Difference	Reduction (%)	99.9% CI of Mean Difference	p-value
Head									
Hands-only CPR		+ Surgical Mask	396.21	±	547.46				
Hands-only CPR		+ N95	375.26	±	263.07	20.95	5.29	− 30.72 − 72.63	0.182
30:2 CPR FMV			972.56	±	411.34				
30:2 CPR FMV	+ Filter		702.58	±	365.60	269.98	27.76	223.18 − 316.78	< 0.001
30:2 CPR SGA			926.73	±	404.22				
30:2 CPR SGA		+ Surgical Mask	942.29	±	421.60	− 15.57	− 1.68	− 65.24 − 34.11	0.302
30:2 CPR SGA			926.73	±	404.22				
30:2 CPR SGA	+ Filter		717.89	±	1717.68	208.84	22.54	58.65 − 359.03	< 0.001
30:2 CPR SGA			926.73	±	404.22				
30:2 CPR SGA	+ Filter	+ Surgical Mask	696.41	±	1138.84	230.32	24.85	127.49 − 333.15	< 0.001
30:2 CPR SGA	+ Filter		717.89	±	1717.68				
30:2 CPR SGA	+ Filter	+ Surgical Mask	696.41	±	1138.84	21.48	2.99	-153.82 − 196.77	0.687
30:2 CPR SGA*	+ Filter		908.76	±	409.39				
30:2 CPR SGA	+ Filter		717.89	±	1717.68	190.87	21.00	40.58 − 341.16	< 0.001
30:2 CPR SGA*	+ Filter	+ Surgical Mask	1020.16	±	765.11				
30:2 CPR SGA	+ Filter	+ Surgical Mask	696.41	±	1138.84	323.75	31.74	207.06 − 440.45	< 0.001
Continuous CPR + ET			780.26	±	320.78				
Continuous CPR + ET		+ Surgical Mask	805.48	±	359.06	− 25.22	− 3.23	− 66.17 − 15.73	0.043
Continuous CPR + ET			780.26	±	320.78				
Continuous CPR + ET	+ Filter		324.30	±	157.79	455.96	58.44	425.55 − 486.38	< 0.001
Continuous CPR + ET			780.26	±	320.78				
Continuous CPR + ET	+ Filter	+ Surgical Mask	213.31	±	108.00	566.95	72.66	538.14 − 595.75	< 0.001
Trunk									
Hands-only CPR		+ Surgical Mask	399.01	±	154.76				
Hands-only CPR		+ N95	190.84	±	76.38	208.17	52.17	193.48 − 222.85	< 0.001
30:2 CPR FMV			942.76	±	384.95				
30:2 CPR FMV	+ Filter		732.05	±	350.33	210.71	22.35	166.44 − 254.98	< 0.001
30:2 CPR SGA			749.36	±	341.98				
30:2 CPR SGA		+ Surgical Mask	961.31	±	634.39	− 211.95	− 28.28	− 273.26 to − 150.64	< 0.001
30:2 CPR SGA			749.36	±	341.98				
30:2 CPR SGA	+ Filter		479.07	±	979.05	270.29	36.07	182.04 − 358.54	< 0.001
30:2 CPR SGA			749.36	±	341.98				
30:2 CPR SGA	+ Filter	+ Surgical Mask	385.67	±	243.00	363.69	48.53	328.01 − 399.37	< 0.001
30:2 CPR SGA	+ Filter		479.07	±	979.05				
30:2 CPR SGA	+ Filter	+ Surgical Mask	385.67	±	243.00	93.40	19.50	7.54 − 179.25	< 0.001
30:2 CPR SGA*	+ Filter		880.15	±	339.51				
30:2 CPR SGA	+ Filter		479.07	±	979.05	401.08	45.57	312.90 − 489.26	< 0.001
30:2 CPR SGA*	+ Filter	+ Surgical Mask	981.86	±	776.33				
30:2 CPR SGA	+ Filter	+ Surgical Mask	385.67	±	243.00	596.19	60.72	526.96 − 665.42	< 0.001
Continuous CPR + ET			767.18	±	328.17				
Continuous CPR + ET		+ Surgical Mask	768.19	±	319.26	− 1.01	− 0.13	− 39.94 − 37.93	0.923
Continuous CPR + ET			767.18	±	328.17				
Continuous CPR + ET	+ Filter		300.96	±	142.80	466.23	60.77	435.78 − 496.68	< 0.001
Continuous CPR + ET			767.18	±	328.17				
Continuous CPR + ET	+ Filter	+ Surgical Mask	354.97	±	156.96	412.21	53.73	381.27 − 443.16	< 0.001
Feet									
Hands-only CPR		+ Surgical Mask	402.67	±	173.49				
Hands-only CPR		+ N95	296.50	±	308.56	106.17	26.37	76.05 − 136.28	< 0.001
30:2 CPR FMV			1182.33	±	933.32				
30:2 CPR FMV	+ Filter		851.00	±	395.31	331.32	28.02	245.08 − 417.56	< 0.001
30:2 CPR SGA			1295.03	±	775.04				
30:2 CPR SGA		+ Surgical Mask	1307.24	±	804.83	− 12.21	− 0.94	− 107.23 − 82.82	0.672
30:2 CPR SGA			1295.03	±	775.04				
30:2 CPR SGA	+ Filter		479.26	±	309.01	815.77	62.99	744.77 − 886.76	< 0.001
30:2 CPR SGA			1295.03	±	775.04				
30:2 CPR SGA	+ Filter	+ Surgical Mask	432.22	±	235.35	862.81	66.62	793.88 − 931.74	< 0.001
30:2 CPR SGA	+ Filter		479.26	±	309.01				
30:2 CPR SGA	+ Filter	+ Surgical Mask	432.22	±	235.35	47.04	9.82	14.01 − 80.08	< 0.001
30:2 CPR SGA*	+ Filter		1300.08	±	679.48				
30:2 CPR SGA	+ Filter		479.26	±	309.01	820.81	63.14	757.30 − 884.32	< 0.001
30:2 CPR SGA*	+ Filter	+ Surgical Mask	1150.06	±	826.29				
30:2 CPR SGA	+ Filter	+ Surgical Mask	432.22	±	235.35	717.84	62.42	644.72 − 790.96	< 0.001
Continuous CPR + ET			1315.70	±	521.18				
Continuous CPR + ET		+ Surgical Mask	1447.51	±	562.61	− 131.80	− 10.02	− 197.03 to − 66.58	< 0.001
Continuous CPR + ET			1315.70	±	521.18				
Continuous CPR + ET	+ Filter		348.80	±	229.02	966.90	73.49	918.47 − 1015.34	< 0.001
Continuous CPR + ET			1315.70	±	521.18				
Continuous CPR + ET	+ Filter	+ Surgical Mask	375.93	±	236.74	939.78	71.43	891.08 − 988.48	< 0.001

ppm, Parts per million; CPR, Cardiopulmonary compression; ET, Endotracheal tube; Filter, High efficiency particulate air filter; FMV, Face mask ventilation; SGA, Supraglottic airway.

*loose-fitting SGA; CI, Confidence interval.

When evaluating HEPA filter effectiveness with 30:2 CPR and FMV, the HEPA filter reduced aerosol concentration 27.76% at the head, 22.35% at the trunk, and 28.02% at the feet, with *p*-values all < 0.001 (Table 2). When HEPA filters were used with tightly-sealed SGA during 30:2 CPR compared to without, the HEPA filter reduced aerosol concentration 22.54% at the head, 36.07% at the trunk, and 62.99% at the feet, with *p*-values all < 0.001 (Table 2). When the HEPA filter was connected to the ET during continuous CPR, the HEPA filter decreased aerosols by 58.44% at the head; by 60.77% at the trunk; and by 73.49% at the feet (all *p*-values < 0.001).

## Discussion

Aerosols containing viable virus can spread disease for hours to days, especially in a poor-ventilated space. Consensus statements agree on donning personal protective device and avoiding aerosolization during chest compressions^23–26^. However, few articles have investigated the effectiveness of current guideline in avoiding aerosol transmission. Public concerns about disease transmission during CPR decreased the willingness of bystanders to perform CPR and may have been related to excess mortality during the height of the COVID-19 pandemic^2,4,6–9,13,14^.

Personnel being in different locations during CPR lasting minutes to hours can expose them to different level of aerosols^19^. According to the present study, the leader or the person administering medication who stands at the feet of the patient may be exposed to more aerosols compared to the airway management personnel and the chest compressor at the head and trunk of the patient during the CPR while applying face mask ventilation (FMV) or a loose-fitting supraglottic airway (SGA), whether using a face mask, High-Efficiency Particulate Air (HEPA) filter or not (Table 1).

Covering the mannequin’s face with a proper fit surgical mask or N95 mask effectively reduced aerosol exposure at the head of the mannequin during hands-only CPR. These findings are compatible with a simulation and cadaver study conducted by Ott et al.^27^ The reduction rate, as measured by the mean difference between the intervention and hands-only CPR without a face covering, were 82.45% and 83.38% at the head, 54.39% and 78.19% at the trunk, and 45.91% and 60.17% at the foot (Table 1). However, the protective effect is surprisingly comparable with CPR when used with endotracheal tube (ET) ventilation and HEPA filters (Table 1). Non-rebreather mask oxygenation at a flow rate of 15 L/min showed a mask effect and reduced the aerosol exposure at the head, trunk, and foot of the mannequin and could be an alternative way if manual ventilation was concerned. This finding is compatible with our previous investigation^19^.

During the 30:2 compression-ventilation ratio CPR, using FMV with a HEPA filter continuously placed on the mannequin’s face reduced aerosol concentrations by 27.76% at the head, 22.35% at the trunk, and 28.02% at the feet (all *p*-values < 0.001) (Table 2). However, using FMV during 30:2 CPR may increase the aerosol exposure to the personnel who standing at the foot side of the mannequin and should wear adequate protective gear (Table 1).

SGA insertion with HEPA filters during CPR with a face covering to minimize aerosol generation was suggested among consensus and two simulation studies^23–28^. However, our results indicated that whether using the surgical mask covering, using the HEPA filter, or both, on 30:2 CPR with a loose-fitting SGA, all settings could not decreased aerosol exposure at the trunk and feet, thus, may increase the risk of infection for the chest compressor and the personnel standing at the foot side(Table 1). We also found significant air leaks under the particle in velocity flow field (Fig. 2). SGA detachment is a risk and, once unveiled, aerosols may be dispersed when the SGA is dragged by the ventilation bag during CPR. This may not be easily detected and could be common in the pre-hospital and in-hospital settings before a definite airway was built. The mask covering or a HEPA filter does not provide any protection if the SGA is not tightly-sealed (Table 2). A properly-fitting SGA should be chosen and kept continuously sealed to the glottis during CPR at all times especially during transportation. When the filter was connected to the ET during continuous CPR, it decreased aerosols by 58.44% without mask and 72.66% with a mask at the head; by 60.77% without a mask and 53.73% with a mask at the trunk; by 73.49% without a mask and 71.43% with a mask at the feet (all *p*-values < 0.001). The face mask covering, when used in combination with oxygenation or ventilation devices, is useless due to loose-fitting (Table 2).

**Figure 3:**
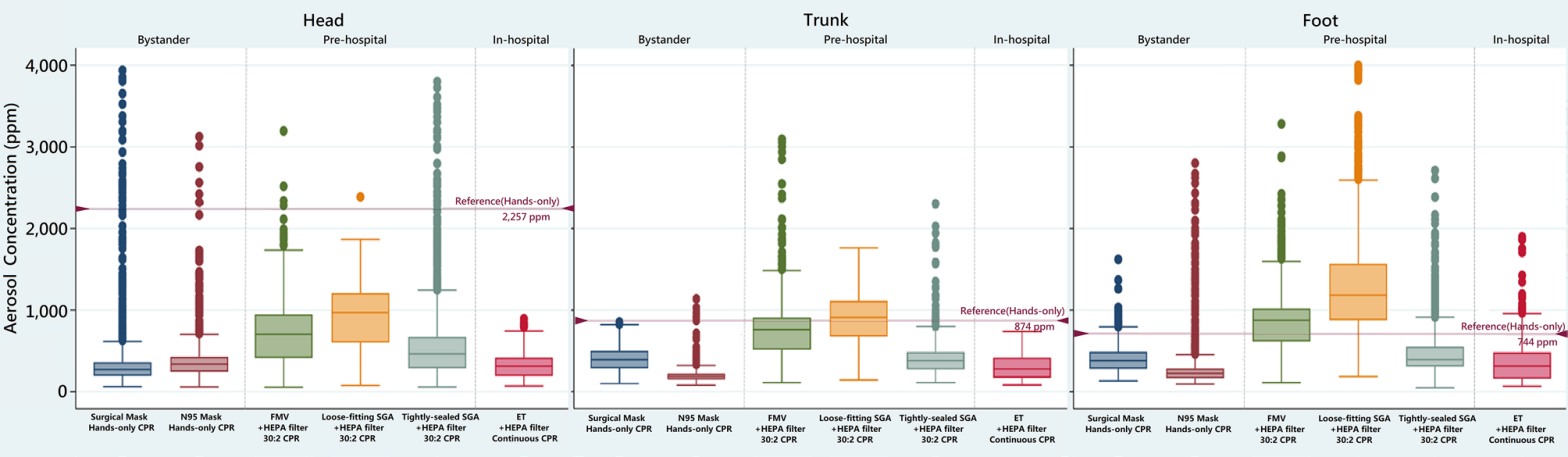
A box plot of aerosol concentrations under the aerosol prevention strategies of bystander, pre-hospital, and in-hospital CPR: under the hands-only CPR plus surgical mask (Mask) covering (bystander CPR); under 30:2 compression–ventilation ratio CPR with the face mask ventilation (FMV) plus a high efficiency particulate air (HEPA) filter (prehospital CPR with FMV); under 30:2 CPR with supraglottic airway (SGA) plus a HEPA filter and a surgical mask (pre-hospital CPR with a loose-fitting SGA); under 30:2 CPR with SGA plus a HEPA filter and a surgical mask (pre-hospital CPR with a tightly-sealed SGA); under continuous CPR with an endotracheal tube (ET) plus a HEPA filter and a surgical mask (in-hospital CPR). Compared with the reference group (red line), all prevention strategies reduced the aerosol exposure at the head of the mannequin in bystander, pre-hospital, and in-hospital settings. In the pre-hospital settings, the FMV with a HEPA filter and the loose-fitting SGA with a HEPA filter and a surgical mask increased the aerosol exposure at the trunk and feet of the mannequin. The tightly-sealed SGA with a HEPA filter and a surgical mask covering, and the ET tube with a HEPA filter and a surgical mask reduced the aerosol exposure at the head, trunk, and feet of the mannequin.

In short, the bioaerosols are sensitive to flow. A tightly-sealed interface between the mask and the victim’s face during the bystander hands-only CPR, or an ET with an inflated balloon tightly sealed with the victim’s trachea and connected to HEPA filters in the in-hospital setting, can safely prevent aerosol dispersion compared with NRM oxygenation, FMV, SGA ventilation during CPR (Fig. 3). Using FMV or SGA ventilation in the pre-hospital setting should choose a very proper size and focus on sealing the ventilation device to the victim’s airway and connect to HEPA filters all the time to avoid aerosol dispersion.

### Limitations

1. The surgical and N95 masks were tested and adequately fit the mannequin’s face before the study. A loose-fitting mask may not generate a similar protective effect.
2. The aerosol movement is highly influenced by the inconstant air flow and humidity in the outdoor environment. This was an indoor simulation study focused on evaluating the risk and current strategies to minimize aerosol dispersion during adult CPR, which may not be indicative of the outdoor environment. However, 76% of OHCA occurred at home or an indoor environment as the hospital^30^. We simulated chest compressions on a mannequin using a central processing unit and a closed pump system connected to a smoke generator to create a constant passive exhaled airflow at a rate of 110 times per minute or a 30:2 compression-ventilation ratio CPR.

The SARS-CoV-2 viral aerosols may disperse differently due to differences in indoor and outdoor airflow, the temperature, and the relative humidity. The viability of SARS-CoV-2 may be different depending on the local environment.

## Conclusion

Surgical or N95 mask face coverings can significantly reduce aerosol exposure during bystander CPR and is quite safe if the mask is properly fit. The aerosol protective effect of surgical or N95 mask coverings is similar to the endotracheal tube (ET) ventilation with HEPA filters. The non-rebreather mask (NRM) oxygenation at 15 L/min and tightly-sealed FMV with filters to the patient’s face during CPR can be an effective alternative strategy. The chest compressor and the personnel standing at the victim’s feet are at a greater risk of exposure if the supraglottic airway (SGA) is not tightly-sealed during 30:2 CPR, even when using High-Efficiency Particulate Air (HEPA) filters. Keeping the SGA tightly sealed at all times is essential to avoid aerosol dispersion. Current strategies to minimize aerosol dispersion during CPR is promising. More education is necessary to reassure the public.

## Supplementary Information

Supplementary Information.

## Data Availability

All data generated or analyzed during this study are included in this published article and its supplementary files.
